# Triple Atresia, Triple Threat? An Unusual Constellation of Primary Surgical Abnormalities

**DOI:** 10.3390/pediatric13020026

**Published:** 2021-04-12

**Authors:** Raef Jackson, Carmen Francis, Karim Awad, Semiu E. Folaranmi

**Affiliations:** 1 Department of Paediatric Surgery, Children’s Hospital for Wales, Cardiff CF14 4XW, UK; carmen.francis@wales.nhs.uk (C.F.); karim.awad@wales.nhs.uk (K.A.); eniola.folaranmi@wales.nhs.uk (S.E.F.); 2 Department of Pediatric Surgery, Ain Shams University Hospitals, Cairo, Egypt

**Keywords:** tracheo-oesophageal fistula with oesophageal atresia (TOF/OA), duodenal atresia (DA), ano-rectal malformation (ARM), VACTERL/VATER association, triple atresia

## Abstract

We present a case series of two patients with tracheo-oesophageal fistula with oesophageal atresia (TOF/OA), duodenal atresia (DA) and ano-rectal malformation (ARM). This constellation of abnormalities, dubbed triple atresia (TA), is a rare combination with few described cases in the literature. Here we describe our management of these cases, as well as the results of our literature review. Both of our cases had staged surgical procedures and were initially managed with thoracotomy for repair of TOF/OA on day two of life. They subsequently underwent laparotomy for management of their abdominal pathology at day five and seven of life. Both have survived the neonatal period and are awaiting definitive surgery for ARM. Literature review yielded seven cases of TA involving a TOF, DA, and ARM. Four patients underwent staged repair, while three patients underwent repair of TOF/OA, DA and colostomy for ARM at the same time. Of these three patients, two died, representing 22% of the overall cohort. Triple atresia remains a rare subset of patients suspected to have VACTERL association, however mortality may be significantly higher. Our data would suggest a staged approach to be optimal for long term survival.

## 1. Introduction

Multiple associations of congenital defects have been described, and perhaps none more so than the VACTERL association. At the forefront of most paediatric surgeons’ minds, this association helps guide our decision making and choice of investigations when assessing babies with tracheo-oesophageal fistula with oesophageal atresia (OA/TOF), as well as those with anorectal malformations (ARM). Further intestinal atresias at either the duodenum (duodenal atresia, DA), jejunum, and ileum have also been described in association with OA/TOF and ARM, however there is a paucity of cases in the literature of triple atresia (TA)—all three anomalies occurring together.

VACTERL association (V—vertebral, A—anorectal, C—cardiac, TE—tracheo-esophageal fistula with oesophageal atresia, R—renal defects, L—radial limb dysplasia) is defined as the presence of three or more of its constituent anomalies, in the absence of other, often similar, associations or syndromes. It is most often diagnosed clinically, where the presence of one or two anomalies will prompt the clinician to further investigate—typically with echocardiography, vertebral X-rays and renal ultrasonography. OA/TOF has been reported as occurring in up to 50–80% of VACTERL patients, and similarly, ARM occurs in approximately 55–90% of cases [1].

Whilst not classically associated with VACTERL, duodenal atresias have been described in patients presenting with a collection of abnormalities in keeping with VACTERL—a retrospective review by Choudhry et al. [2] found the incidence of VACTERL in cases of prenatally diagnosed DA to be 5%, in addition to isolated case reports [3].

We describe a case series of two infants born with triple atresia (TA), that is—an infant found with simultaneous OA/TOF, DA or other intestinal atresia, and an ARM—our management of these infants-, short- and long-term outcomes, as well as our review of the literature of this uncommon constellation of primary surgical abnormalities. 

## 2. Case Reports

Our first case is of a singleton baby boy born at 31 + 3 weeks weighing 1.7 kg at birth. Antenatal anomaly scans were significant for an atrio-ventricular septal defect, as well as a multi-cystic dysplastic right kidney. The child was born to non-consanguineous parents with a history of maternal smoking. The antenatal comparative genomic hybridisation (CGH) array was normal, as was the postnatal whole genome sequence. The paternal first cousin had a history of tetralogy of Fallot and an associated gene microdeletion, there was no further family history of note. 

The infant was delivered via normal vaginal delivery following spontaneous rupture of membranes at a peripheral hospital. The child was reportedly in poor condition, cyanosed, with no spontaneous respiratory effort requiring inflation breaths and early intubation, stabilizing with a PEEP at 6 cm H_2_O and FiO_2_ at 40%. Initial examination demonstrated an absent anal opening, and an NG tube could not be advanced into the stomach following endotracheal intubation. Radiographs demonstrated coiling of the NG tube in the upper mediastinum, as well as the appearance of a double-bubble gas pattern in the abdomen. The child was promptly transferred to our hospital. 

Postnatal echocardiography demonstrated a left-sided aortic arch, large PDA with bi-directional flow, a high medium-sized secundum ASD as well as normal variant left SVC draining to the coronary sinus—cardiology opinion was sought, and the child’s cardiac anomalies were not found to be significant enough to delay theatre. Post-natal renal ultrasonography confirmed right multi-cystic dysplastic kidney.

The combination of TOF/OA, ARM, renal and cardiac abnormalities were highly suggestive of VACTERL association. The diagnosis was confirmed when no additional features suggestive of an alternative diagnosis were identified.

The infant was taken to theatre on day two of life for direct laryngotracheobronchoscopy (DLTB), thoracotomy and primary repair of OA/TOF. DLTB demonstrated a distal TOF, with no evidence of additional airway anomalies or an upper pouch fistula. Thoracotomy findings were of a type C atresia (oesophageal atresia with distal trachea-oesophageal fistula) with a relatively wide gap (three vertebral bodies). This was repaired in standard fashion, by performing an end-to-end anastomosis, under some tension, utilizing interrupted 6–0 PDS sutures over a 6 Fr trans-anastomotic tube (TAT). An 8 Fr chest drain was left in situ.

The child remained clinically stable post-operatively, remaining intubated and on conventional ventilation, whilst being fed exclusively parenterally. Returning to theatre on day 7 of life, the child underwent laparotomy, repair of duodenal atresia, and fashioning of a distal colostomy which was sited in the medial edge of the right transverse incision. Intra-operative findings were of a type 3 duodenal atresia, mobile caecum, wide small bowel mesentery with normal rotation, and a large right sided retroperitoneal cyst, in keeping with a MCDK. We performed a standard Kimura duodeno-duodenostomy, advancing the TAT past the site of the duodenal anastomosis, as well as a split sigmoid colostomy. The retroperitoneal cyst was aspirated to aid in abdominal wall closure. Standard anaesthetic monitoring was utilized for both procedures with no reported anaesthetic complications.

The child progressed well post-operatively, started on enteral feeds via the TAT and reached full feeds on day 27 of life. He was extubated on day 21 and placed on BiPAP. He subsequently moved onto CPAP on day 31, and high-flow nasal cannulae on day 78. The delay in progression in terms of his respiratory support was partially explained by his PDA, which remained haemodynamically significant throughout his stay in hospital. He was discharged on day 90 of life, and subsequently underwent coil occlusion of PDA on day 158 of life. 

**Figure 1:**
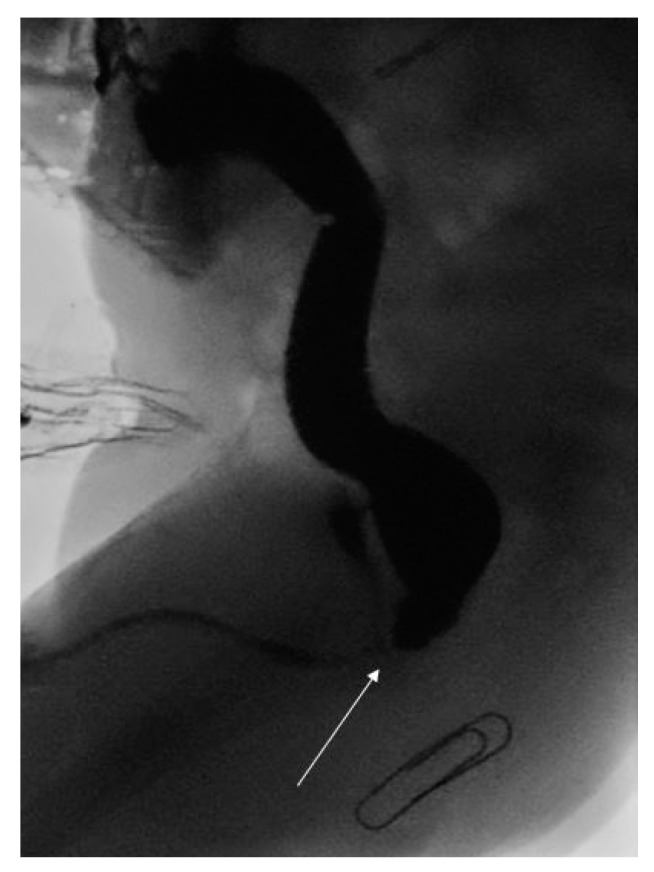
High pressure distal loopogram demonstrating recto-bulbar fistula (white arrow) and expected position of neo-anus (paper clip).

Following cardiac surgery, the child underwent a high pressure distal loopogram that confirmed a recto-bulbar ARM (see Figure 1). This was managed with a posterior sagital anorectoplasty (PSARP) on day 313 of life. Interestingly, due to the high position of the original stoma in the medial edge of the right transverse incision, the stoma had to be re-sited to avoid undue tension across the colo-anal anastamosis. He went on to have an uncomplicated post-operative recovery and remained well at one month follow up.

Our second case is of a term baby girl born at 37 weeks gestation weighing 2.4 kg. The child had an antenatal history of polyhydramnios and a double-bubble on ultrasound, however amniocentesis did not demonstrate any chromosomal abnormalities. There was no significant family history of note. 

The child was born via emergency caesarean due to a non-reassuring cardiotocography (CTG) finding. Resuscitation was required for up to two minutes of life, and during these efforts it was noted that an NG was unable to pass despite multiple attempts; a replogle tube was therefore sited to the upper oesophageal pouch. Due to ongoing respiratory distress, the child was intubated. 

**Figure 2:**
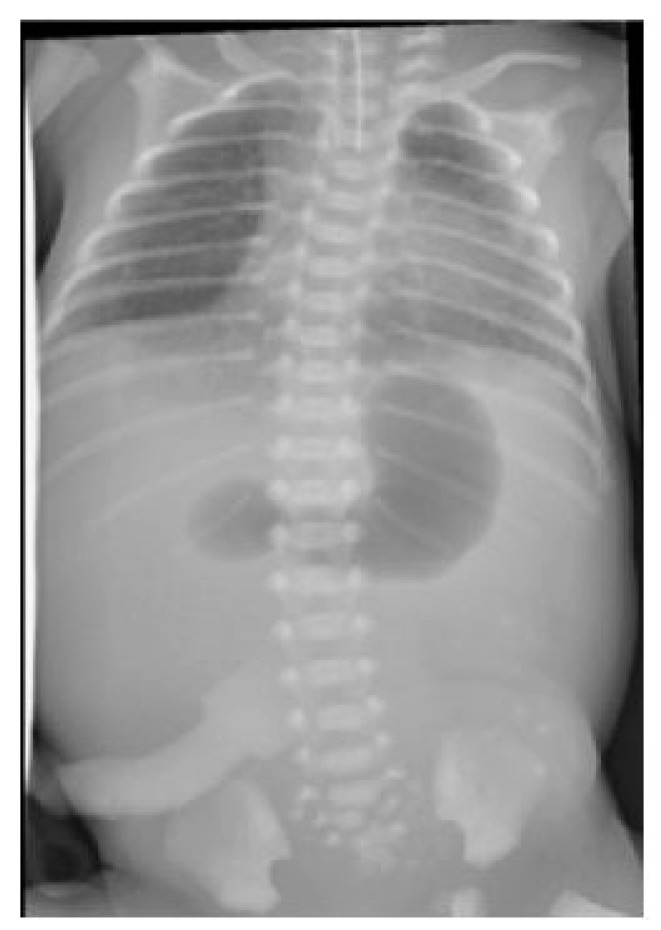
Radiograph demonstrating characteristic double-bubble appearance.

An abdominal and chest radiograph confirmed the diagnosis of oesophageal atresia and tracheo-oesophageal fistula, as well as a concurrent duodenal atresia (see Figure 2). Further examination of the neonate demonstrated flat buttocks and an anorectal malformation with recto-vestibular fistula; no additional abnormalities were identified. 

The infant was taken to theatre on day two of life for direct laryngotracheobronchoscopy (DLTB), thoracotomy and primary repair of OA/TOF. Bronchoscopy findings were of a distal fistula only, and at thoracotomy a type C OA/TOF was identified. The fistula was ligated and a primary oesophageal anastomosis was performed. 

The infant returned to theatre on day four of life for laparotomy and duodeno-duodenostomy; intra-operative findings were of an annular pancreas. Examination of the perineum demonstrated a fistula which calibrated to a size 10 hegar, thus a stoma was not formed. Standard anaesthetic monitoring was utilized for both procedures with no reported anaesthetic complications. 

Extubation was achieved five days post laparotomy. The infant initially made a good recovery, however she then developed a pleural effusion which was confirmed to be chyle and therefore had a chest drain insertion. This was managed conservatively by commencing monogen enterally, supported by parenteral nutrition (PN) and octreotide, with resolution on day 53 of life. EBM (expressed breast milk) was then commenced and full feeds achieved on day 68 of life. 

Following discharge home, the infant underwent an uncomplicated anorectoplasty on day 141 of life. She remained well at follow up one month post discharge.

## 3. Results

To further assess previously described cases of triple atresia, we conducted a review of the literature utilizing relevant search terms, yielding an initial 26 articles. These were reviewed individually with 23 excluded as they did not report cases of triple atresia. The three remaining articles described a total of seven cases, which are outlined in Table 1 [4,5,6].

Among the seven previously described cases, there were five males representing 71% of the group. All were diagnosed within four days of birth. The primary anomaly was an OA/TOF in in six cases, and a pure oesophageal atresia in one. The primary intestinal pathology was duodenal atresia in five cases, annular pancreas in one, and a pyloric web in one. All seven cases were reported to have an “absent anal opening”. 

Four cases underwent staged repair of their respective anomalies, with all four of these cases from the same institution. Of these four, all underwent laparotomy and repair of their intestinal pathology with a distal colostomy before thoracotomy, ligation of TOF and OA repair, which on average was performed 72 h after laparotomy. All seven cases had sigmoid colostomies performed with a plan for subsequent repair of the ARM in the non-acute phase. 

Overall two infants in the previously described cases died, representing a combined mortality rate of 22%. Interestingly, the two infants who passed away both underwent single-stage procedures (combined laparotomy and thoracotomy). 

## 4. Discussion

Triple atresia remains a complex and challenging combination of anomalies to treat. With few cases in the literature it can be difficult to draw precise conclusions regarding optimal management and surgical approaches; however a few key principles are suggested here.

One could argue the most pressing question for the clinician facing a case of triple atresia would be the timing and sequence of surgical intervention, whether to utilize a combined or staged approach, in addition to the likely prognosis for the infant. 

In all cases that we present, infants were first operated on within four days and on average within 60 h. We recommend operating on an emergent basis following stabilization of the child would be most appropriate in these cases. The question as to whether to proceed with thoracotomy and management of OA/TOF before laparotomy and management of intestinal pathology remains controversial—the dangers of a ventilated OA/TOF have been described in the literature previously, however recent evidence presented by Yardley et al. [7] would suggest that these risks may be overstated. We chose to operate on the OA/TOF before performing the laparotomy in order to avoid the risk of the infant deteriorating while being ventilated with a trachea-oesophageal fistula. In our cases intubation and ventilation occurred shortly after birth and (in one case) prior to transfer to our centre, with an associated time lag. We do note, however, that four cases in our case series had ligation of TOF two to four days following initial laparotomy [6] and intubation; though successful in these cases, we would suggest that the risks of keeping an infant with a proven TOF ventilated outweigh the negligible benefits of performing a laparotomy as the primary procedure and would therefore recommend a thoracotomy first approach. 

The data we present here indicates an overall mortality rate of 22%. While case numbers here are low, this remains consistent with reported mortality in previous series for infants born with OA/TOF and DA, without ARM—suggesting no additional mortality burden with the additional anorectal pathology [1,8]. Interestingly, the cases of mortality occurred when a combined thoracotomy and laparotomy were performed, whereas all the survivors underwent a staged approach. This higher mortality rate with a combined vs staged approach has also been reported previously—Ein et al. [8] reported a survival rate of 83% vs. 57% with staged vs combined approaches to OA/TOF and DA—however there were significant reported differences between these groups, namely respiratory distress and delayed diagnoses, that could also explain the results.

## 5. Conclusions

In summary, there are many uncertainties in the management of these complex neonatal cases. Our case series and literature review would suggest that a staged approach would confer a survival advantage, however as previously stated the reported case numbers for this combination of conditions remains extremely low and it therefore remains challenging to make definitive recommendations. Further experience and reporting of these anomalies, when they occur, would bolster our understanding of the optimal management of triple atresia.

## Figures and Tables

**Table 1:** Summary of literature review.

Author	Case Description	First Operation	Second Operation	Third Operation	Follow Up
**Panda 2015**	2 day old male abdominal distension, drooling, ARM (1) OA/TOF on XR with NG failing to advance (2) DA on XR double-bubble (3) Absent anal opening	Sigmoid colostomy + D-D (type 1 DA)	After 72 h Primary repair of TOF/OA	After 12 months PSARP followed by colostomy closure	Reviewed at 3 years of age, no major concerns.
	4 day old female, drooling, no meconuria, anuria (1) OA/TOF on XR with NG failing to advance (2) Annular pancreas found at laparotomy (3) Absent anal opening	Excision of type IV pouch colon + repair of colovesicular fistula + end colostomy	After 72 h Division of TOF and cervico- and abdominal esophagostomy	After 96 h Duodeno-duodenostomy (annular pancreas)	Planned oesophageal replacement surgery and ARM repair
	3 day old male drooling, abdominal distension (1) OA/TOF on XR—free gas suspicious of perforation (2) DA found after starting feeds on day 9 (3) Absent anal opening	Repair of posterior stomach wall perforation, gastrostomy, and sigmoid colostomy	After 48 h TOF/OA repair	After 9 days Lateral duodenotomy and incision of duodenal web	Subsequently went on to have PSARP at 9 months of age. No major concerns at follow up.
	2 day old female, drooling and failure to pass meconium (1) Pure OA—NG failing to advance, no distal gas (2) DA found at laparotomy (3) Absent anal opening	Sigmoid colostomy–repair of colovesicular fistula, cervical oesophagostomy, gastrostomy, Ladd’s procedure and D-D	N/A	N/A	Subsequently went on to have PSARP at 1 year of age. Continues to have fecal soiling secondary to sacral agenesis
	5 day old male, OA/TOF having had a sigmoid colostomy (1) OA/TOF on XR with NG failing to advance (2) DA on XR double-bubble (3) Absent anal opening	Sigmoid colostomy (peripheral hospital)	After 96 h TOF/OA repair	After 72 h Duodeno-duodenostomy	Subsequently went on to have PSARP at 2 years of age. Also required 2 oesophageal dilatations
**Harjai 2000**	1 day old male, ARM and regurgitation after feeds (1) OA/TOF on XR with NG failing to advance (2) DA on XR double-bubble (3) Absent anal opening	TOF/OA repair primary anastomosis + D-D, gastrostomy and sigmoid colostomy	N/A	N/A	Died day 8—anastamotic leak from TOF/OA
**Khanna 2017**	2 day old male, drooling and unable to pass NG. (1) OA/TOF on XR with NG failing to advance (2) Pyloric web (3) Absent anal opening	TOF/OA repair, primary anastomosis + laparotomy, H-M pyloroplasty for pyloric web, sigmoid colostomy.	N/A	N/A	Died POD 6—sepsis, pneumonia

TOF/OA—tracheo-oesophageal fistula with oesophageal atresia, NG—naso-gastric, DA—duodenal atresia, XR—X-rays, PSARP—Posterior saggital ano-rectoplasty, ARM—anorectal malformation, D-D–Duodeno-duodenostomy.

## Data Availability

Not applicable.
